# Nucleophosmin 1 is a prognostic marker of gastrointestinal cancer and is associated with m6A and cuproptosis

**DOI:** 10.3389/fphar.2022.1010879

**Published:** 2022-09-14

**Authors:** Xu-Sheng Liu, Chao Liu, Jing Zeng, Dao-Bing Zeng, Yi-Jia Chen, Fan Tan, Yan Gao, Xiao-Yu Liu, Yu Zhang, Yao-Hua Zhang, Zhi-Jun Pei

**Affiliations:** ^1^ Department of Nuclear Medicine, Taihe Hospital, Hubei University of Medicine, Shiyan, China; ^2^ Medical Imaging Center, Taihe Hospital, Hubei University of Medicine, Shiyan, China; ^3^ Department of Infection Control, Taihe Hospital, Hubei University of Medicine, Shiyan, China; ^4^ Hubei Clinical Research Center for Precise Diagnosis and Treatment of Liver Cancer, Taihe Hospital, Hubei University of Medicine, Shiyan, China

**Keywords:** NPM1, gastrointestinal cancer, immune infiltrates, cuproptosis, m6A

## Abstract

**Background:** NPM1 is highly expressed in a variety of solid tumors and promotes tumor development. However, there are few comprehensive studies on NPM1 analysis in gastrointestinal cancer.

**Methods:** We used bioinformatics tools to study the expression difference of NPM1 between gastrointestinal cancer and control group, and analyzed the relationship between its expression level and the diagnosis, prognosis, functional signaling pathway, immune infiltration, m6A and cuproptosis related genes of gastrointestinal cancer. At the same time, the expression difference of NPM1 between esophageal carcinoma (ESCA) samples and control samples was verified by *in vitro* experiments.

**Results:** NPM1 was overexpressed in gastrointestinal cancer. *In vitro* experiments confirmed that the expression of NPM1 in ESCA samples was higher than that in normal samples. The expression of NPM1 has high accuracy in predicting the outcome of gastrointestinal cancer. The expression of NPM1 is closely related to the prognosis of multiple gastrointestinal cancers. Go and KEGG enrichment analysis showed that NPM1 co-expressed genes involved in a variety of biological functions. NPM1 expression is potentially associated with a variety of immune cell infiltration, m6A and cuproptosis related genes in gastrointestinal cancers.

**Conclusion:** NPM1 can be used as a diagnostic and prognostic marker of gastrointestinal cancer, which is related to the immune cell infiltration and the regulation of m6A and cuproptosis.

## Introduction

About 10 million people die of malignant tumors every year in the world, among which gastrointestinal cancer is a common malignant tumor in the digestive system (Sung et al., 2021). Common gastrointestinal cancers include cholangiocarcinoma (CHOL), colon adenocarcinoma (COAD), esophageal carcinoma (ESCA), liver hepatocellular carcinoma (LIHC), pancreatic adenocarcinoma (PAAD), rectum adenocarcinoma (READ) and stomach adenocarcinoma (STAD). Although significant progress has been made in radical resection, radiotherapy and chemotherapy, the 5-years survival rate of patients with gastric cancer is still very low (Shen et al., 2020; Thrift, 2021). The occurrence and development of gastrointestinal cancer is the result of multiple genes and factors. Therefore, seeking diagnostic markers with high sensitivity and specificity is of great significance to improve the diagnostic level of gastrointestinal cancer, especially to improve the diagnostic rate of early cancer and prolong the life of patients.

In the nucleus, nucleophosmin 1 (NPM1) is a multifunctional protein that shuttles between the nucleus and the cytoplasm (Karimi Dermani et al., 2021). In previous studies, NPM1 has mainly been studied in relation to blood system diseases, and only a few reports have been published regarding solid tumors (Zarka et al., 2020; Dong et al., 2022). There are strong relationships between the expression of NPM1 and glycolysis related genes as well as ^18^F-FDG metabolism parameters in lung adenocarcinoma (LUAD) in our previous studies (Liu et al., 2021a; Zhou et al., 2021a). However, the study of NPM1 in gastrointestinal cancer, especially in esophageal cancer, has not been reported.

Many studies have shown that the tumor microenvironment (TME) with extensive immune infiltration and N6-methyladenosine (m6A) modification that regulates gene stability play an important role in the occurrence and development of tumors and the diagnosis and treatment of cancer (Chen et al., 2019; Wang et al., 2020a; Chamma et al., 2022). At the same time, a recently discovered copper dependent regulatory cell death (cuproptosis) has become a research hotspot (Cobine and Brady, 2022; Tang et al., 2022; Tsvetkov et al., 2022). NPM1 has been studied relatively little in gastrointestinal cancer despite its multifaceted nature, specifically on its relationship with immunotherapy, m6A modification, and cuproptosis.

We downloaded gastrointestinal cancer data from The Cancer Genome Atlas (TCGA) for this study. The R software package and other online databases were used to analyze the expression and prognostic value of NPM1 in gastrointestinal cancer. NPM1 expression in ESCA samples was compared to that in normal samples using cell experiments and immunohistochemistry (IHC). Furthermore, NPM1’s function and pathway enrichment in gastrointestinal cancer were examined as well as its co-expression network in gastrointestinal cancer. Finally, NPM1 and tumor immunity, m6A methylation modification, and cuproptosis related genes in ESCA were studied to establish a basis for developing new treatment strategies.

## Materials and methods

### Expression level and prognostic value of nucleophosmin 1 in public database

The expression difference of NPM1 in Pan-cancer was analyzed using UCSC XENA database (https://xenabrowser.net/datapages/) (Vivian et al., 2017). UCSC XENA database contains The Cancer Genome Atlas (TCGA; https://portal.gdc.cancer.gov/) (Tomczak et al., 2015) and Genotype-Tissue Expression (GTEx; http://commonfund.nih.gov/GTEx/) (Battle et al., 2017) data sets, which are mainly used to analyze and visualize gene expression in tumors. We downloaded the cholangiocarcinoma (CHOL), colorectal cancer (CRC), Esophageal carcinoma (ESCA), liver hepatocellular carcinoma (LIHC), pancreatic adenocarcinoma (PAAD) and stomach adenocarcinoma (STAD) data sets from the Gene Expression Omnibus database (GEO; GSE76297, GSE9348, GSE23400, GSE45267, GSE11838 and GSE66229; www.ncbi.nlm.nih.gov/geo) to verify whether NPM1 mRNA expression differs between tumors and controls. A human protein atlas database (HPA; https://www.proteinatlas.org/) immunohistochemistry image of NPM1 was obtained to demonstrate the protein’s expression. The clinical data of CHOL, COAD, ESCA, LIHC, PAAD, READ and STAD data sets were downloaded from TCGA database to study the relationship between the expression level of NPM1 and the prognostic value of patients with gastrointestinal tumors. A ROC curve was drawn to evaluate the diagnostic value of NPM1 in gastrointestinal tumors.

### *In vitro* experiment

A qRT-PCR assay and immunohistochemistry (IHC) staining assay were used to determine whether NPM1 expression differed between tumors and normals. We referred to previous studies for qRT-PCR and IHC experimental steps (Liu et al., 2021b; Liu et al., 2021c). Technical details can be found in the Supplementary Materials.

### Co-expression network analysis of nucleophosmin 1 in gastrointestinal cancer

We used the STAT package in R to analyze RNA sequencing data from TCGA patients with gastrointestinal cancer in order to study co-expressed genes associated with NPM1. For statistical analysis, Pearson correlation coefficient was used. In the case of |cor| > 0.3, *p* < 0.05, we consider the correlation to be significant. Correlation heat map and Veen map are drawn by using ggplot2 software package in R language. There are 30 genes that are positively correlated with NPM1 expression in gastrointestinal cancers, as shown in the correlation heat map. Veen map shows the intersection of genes related to NPM1 expression in gastrointestinal tumors, the threshold is cor >0.3, *p* < 0.05. In order to screen out the 30 genes most related to the expression of NPM1 in gastrointestinal cancer, we summed the correlation coefficients of each gene and sorted them according to the average correlation coefficient, and finally got the 30 most related genes.

### Nucleophosmin 1 co-expression network enrichment analysis

The above intersection of genes related to NPM1 expression were enriched and analyzed, mainly including Gene ontology (GO, http://www.geneontology.org/) and Kyoto Encyclopedia of Genes and Genomes (KEGG, http://www.genome.jp/kegg/). ClusterProfiler package (Yu et al., 2012) of R language was used for enrichment analysis, and ggplot2 package was used for visualization.

### The relationship between nucleophosmin 1 and tumor immune infiltrating cells in gastrointestinal cancers

Tumor Immune Estimation Resource (TIMER, https://cistrome.shinyapps.io/timer) is a comprehensive resource for systematical analysis of immune infiltrates across diverse cancer types (Li et al., 2016; Li et al., 2017). In this study, we used three algorithms in timer database, namely TIMER (22), QUANTISEQ (Finotello et al., 2019) and CIBERSORT(Newman et al., 2015), to determine whether NPM1 correlates with immune cells that contribute to gastrointestinal cancer. Next, we used TISIDB database (http://cis.hku.hk/TISIDB/index.php) to further study the expression level of NPM1 in different immune subtypes. There is a database that contains information about tumors and their interactions with the immune system (Ru et al., 2019).

### Correlation between nucleophosmin 1 with m6A and cuproptosis related genes in gastrointestinal cancer

In order to further study the possibility that NPM1 may be involved in the regulation of m6A and cuproptosis in gastrointestinal cancer, an analysis of NPM1 expression along with m6A and cuproptosis related genes was conducted using the TCGA database. Based on previous studies (Li et al., 2019; Tsvetkov et al., 2022), we collected 20 m6A related genes and 10 cuproptosis related genes. The relationship between NPM1 with m6A and cuproptosis related gene expression in gastrointestinal cancer was analyzed by R software package. Based on the difference in NPM1 expression levels, we divided the TCGA ESCA cohort into two groups and analyzed the difference of m6A and cuproptosis related gene expression between high and low NPM1 expression groups. At the same time, we also analyzed the expression differences of m6A and cuproptosis related genes between the tumor and normal group in the TCGA ESCA cohort. Finally, according to the correlation of expression level and the difference of group expression, we screened the genes most likely to interact with NPM1. Use ggplot2 software package for data visualization.

### Statistical methods

Most statistical analysis is done through the above bioinformatics tools. This includes Xiantao platform (www.xiantao.love). Xiantao platform is a database integrating TCGA tumor chip data, which contains R software and its appropriate R software package. It is mainly used for gene expression analysis, correlation analysis, enrichment analysis, interactive network analysis, clinical significance analysis, and related mapping. The value of *p* < 0.05 was considered statistically significant.

## Results

### Multiple databases verified that nucleophosmin 1 was highly expressed in gastrointestinal cancer

**FIGURE 1:**
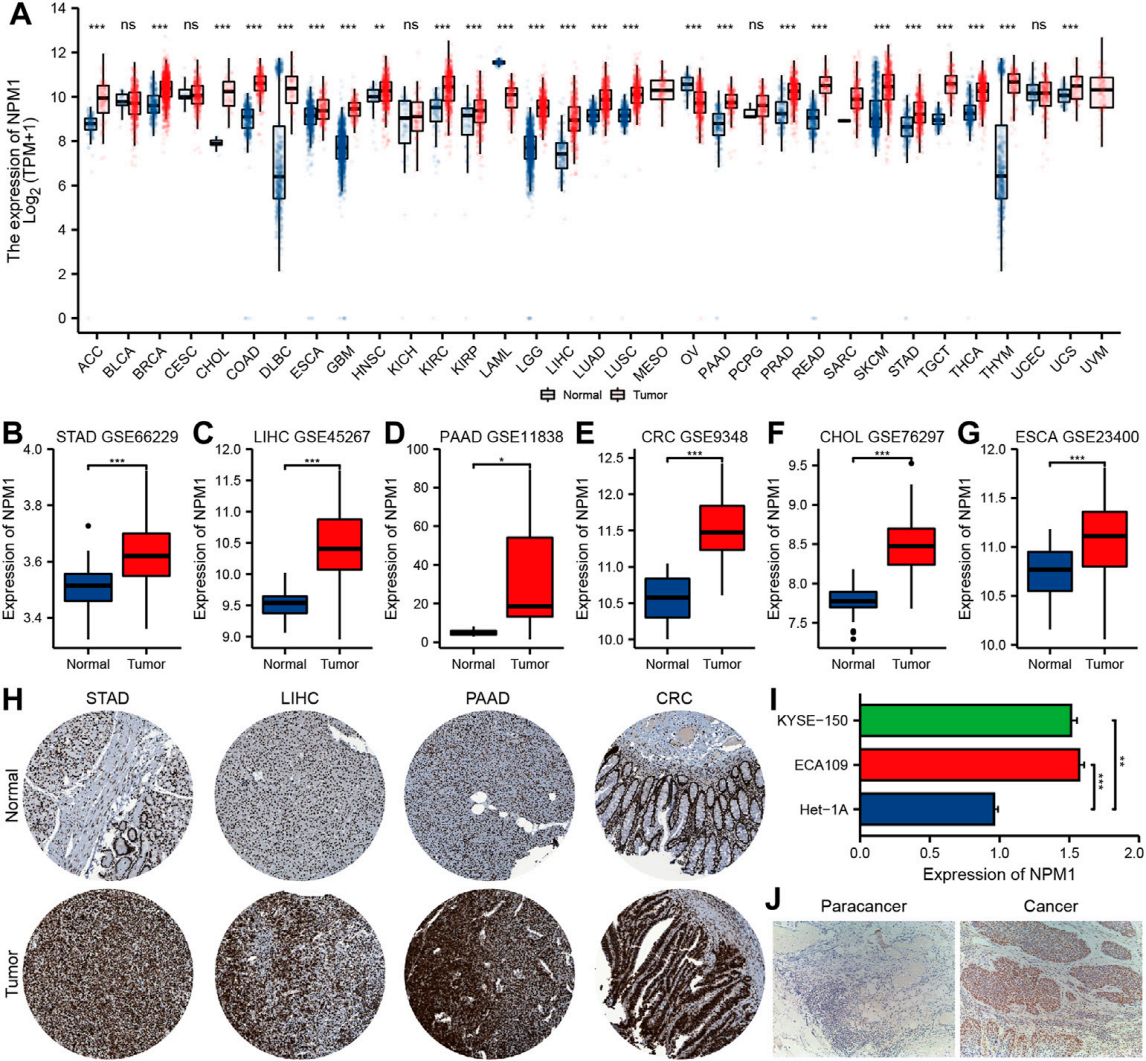
Multiple databases verified that NPM1 was highly expressed in gastrointestinal cancer. **(A)** The analysis of UCSC XENA database showed that NPM1 was highly expressed in a variety of tumor tissues. **(B–G)** According to the GEO database, compared to controls, gastrointestinal cancers expressed more NPM1. **(H)** There was a difference in NPM1 expression between tumor samples and normal samples in the HPA database. **(I)** Cell experiments showed that NPM1 expression was significantly higher in ESCA cells compared to normal esophageal cells. **(J)** According to IHC results, tumor tissues from ESCA patients expressed significantly more NPM1 than paracancer tissues. *p < 0.05; **p < 0.01; ***p < 0.001; ns, no significance.

Based on analysis of the UCSC XENA database, NPM1 expression was higher in ACC (adrenocortical carcinoma), BRCA (breast invasive carcinoma), CHOL, COAD, DLBC (lymphoid neoplasm diffuse large b-cell lymphoma), ESCA, GBM (glioblastoma multiforme), HNSC (head and neck squamous cell carcinoma), KIRC (kidney renal clear cell carcinoma), KIRP (kidney renal papillary cell carcinoma), LGG (brain lower grade glioma), LIHC, LUAD (lung adenocarcinoma), LUSC (lung squamous cell carcinoma), PAAD, PRAD (prostate adenocarcinoma), READ, SKCM (skin cutaneous melanoma), STAD, TGCT (testicular germ cell tumors), THCA (thyroid carcinoma), THYM (thymoma) and UCS (uterine carcinosarcoma) than in controls, but lower in LAML (acute myeloid leukemia) and OV (ovarian serous cystadenocarcinoma) (Figure 1A). GEO database analysis further confirmed that the expression of NPM1 in gastrointestinal cancers were higher than that in normal control group (Figures 1B–G). A HPA database IHC staining revealed a significant increase in NPM1 expression in STAD, LIHC, PAAD, and CRC tissues (Figure 1H).

In addition, qRT-PCR showed that the expression level of NPM1 mRNA in ESCA cell line was significantly higher than that in normal esophageal tissue cells (Figure 1I). A significant difference was observed between tumor tissues of ESCA patients and those of paracancer patients (Figure 1J).

### Prognostic and diagnostic value of nucleophosmin 1 in gastrointestinal cancers

**FIGURE 2:**
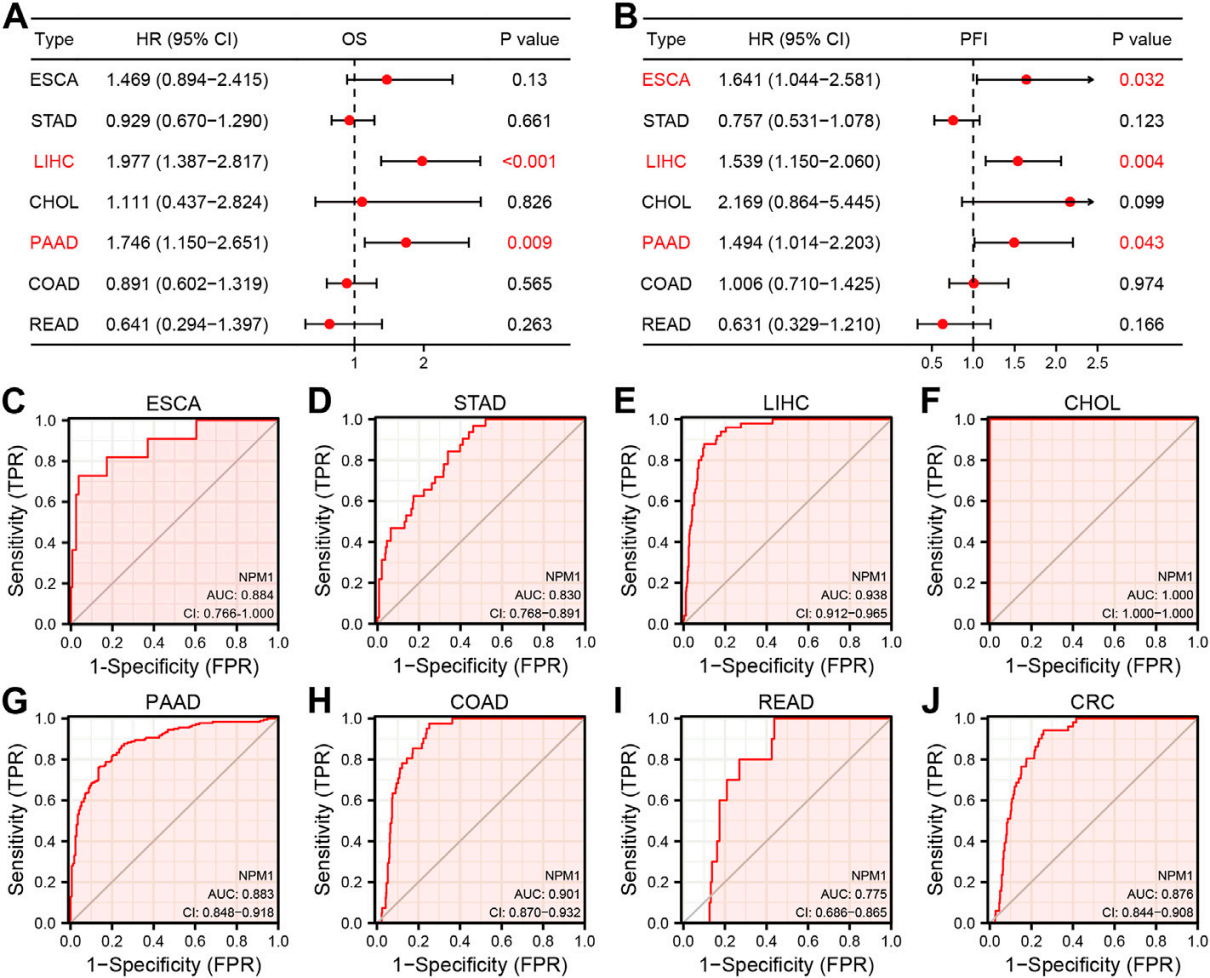
Prognostic and diagnostic value of NPM1 in gastrointestinal cancers. **(A–B)** Forest map shows the relationship between NPM1 expression level with OS and PFI, respectively. **(C–J)** ROC curve shows the value of NPM1 in the diagnosis of patients with gastrointestinal cancer, including ESCA, STAD, LIHC, CHOL, PAAD, COAD, READ and CRC.

In order to further explore the relationship between NPM1 expression and gastrointestinal cancer, we analyzed the relationship between NPM1 expression and pathological characteristics of cancer patients. The results showed that patients with high expression of NPM1 were associated with worse OS in LIHC [HR: 1.977 (1.387–2.817), *p* < 0.001] and PAAD [HR: 1.746 (1.150–2.651), *p* = 0.009]. In ESCA [HR: 1.641 (1.044–2.581), *p* = 0.032], LIHC [HR: 1.539 (1.150–2.060), *p* = 0.004] and PAAD [1.494 (1.014–2.203), *p* = 0.043], PFI was lower in patients with high expression of NPM1. At the same time, we also found that NPM1 had high accuracy in diagnosing ESCA, STAD, LIHC, CHOL, PAAD, COAD, READ, and CRC patients and normal controls (Figure 2, AUC > 0.75).

### Co-expression network analysis of nucleophosmin 1 in gastrointestinal cancer

**FIGURE 3:**
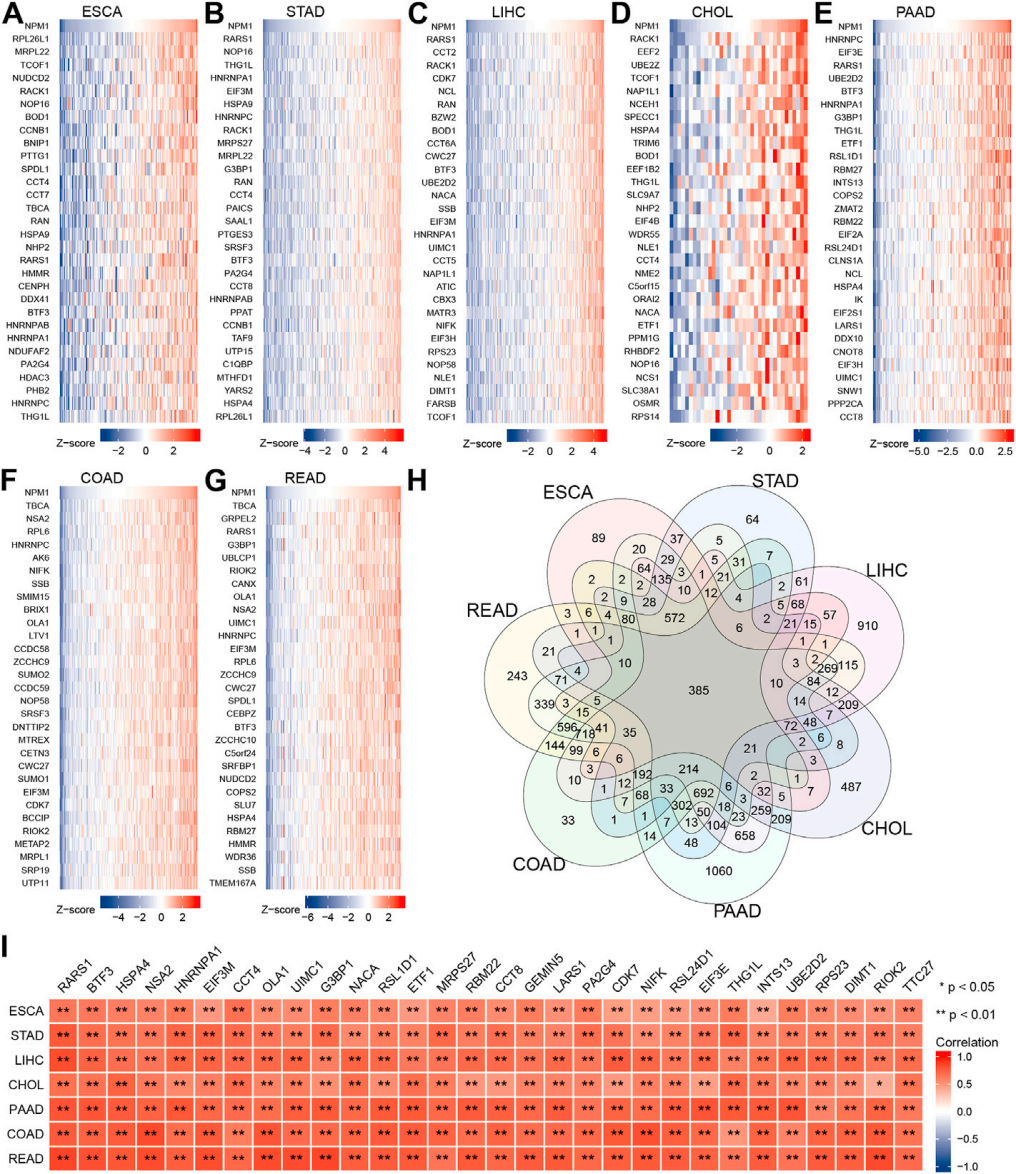
Co-expression network analysis of NPM1 in gastrointestinal cancer. The top 30 genes in ESCA **(A)**, STAD **(B)**, LIHC **(C)**, CHOL **(D)**, PAAD **(E)**, COAD **(F)** and READ **(G)** that are positively linked with NPM1 expression are displayed in a heat map. **(H)** Veen map shows the intersection of these gene sets. **(I)** The related heat map shows the 30 co-expressed genes sorted according to the average correlation coefficient. *p < 0.05; **p < 0.01.

We analyzed RNA sequencing data from the TCGA database of gastrointestinal cancer with R software, and only retained gene encoding proteins from the gene sequences. The analysis found that 1958 genes expression in ESCA were associated with NPM1, 3,302 genes expression in STAD were associated with NPM1, 6,438 genes expression in LIHC were associated with NPM1, 2,628 genes expression in CHOL were associated with NPM1, 7,721 genes expression in PAAD were associated with NPM1, 4,523 genes expression in COAD were associated with NPM1, and 5,602 genes expression in READ were associated with NPM1. |cor| > 0.3 and *p* < 0.05 were the thresholds. The top 30 genes in ESCA, STAD, LIHC, CHOL, PAAD, COAD, and READ that are positively linked with NPM1 expression are displayed in a heat map (Figures 3A–G). Veen map shows the intersection of these gene sets, and 385 genes are found to coincide (Figure 3H). Figure 3I shows the 30 co-expressed genes sorted according to the average correlation coefficient. The top three genes in the average correlation coefficient were RARS1 (Arginyl-TRNA Synthetase 1, average cor = 0.763), BTF3 (Basic Transcription Factor 3, average cor = 0.725) and HSPA4 (Heat Shock Protein Family A (Hsp70) Member 4, average cor = 0.716), respectively.

### Nucleophosmin 1 co-expression network enrichment analysis

**FIGURE 4:**
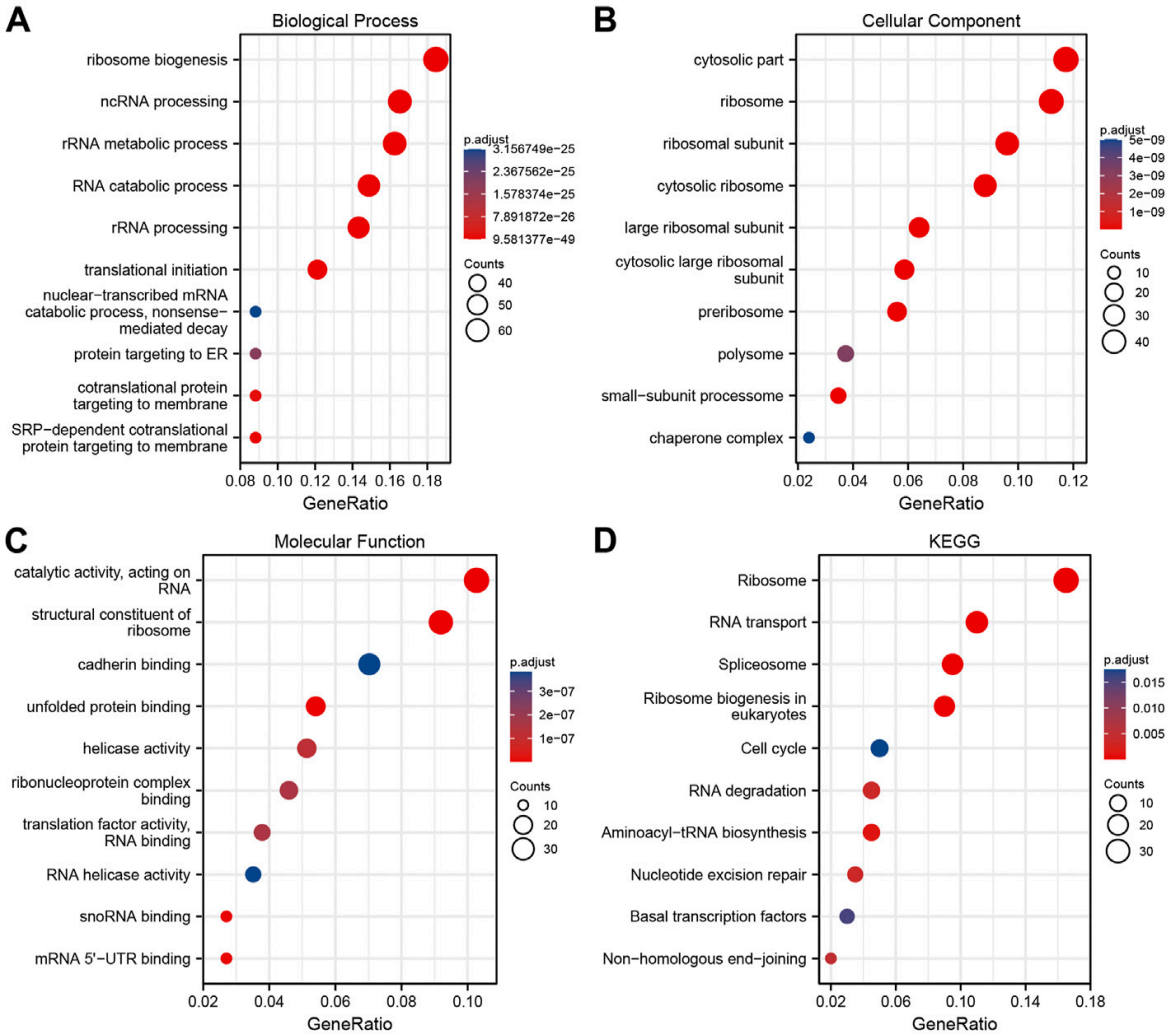
NPM1 co-expression network enrichment Analysis. **(A–C)** An enrichment analysis of GO terms for co-expression genes with NPM1. **(D)** An enrichment analysis of KEGG terms for NPM1 co-expression genes.

ClusterProfiler package in R was used to analyze the above intersection of genes related to NPM1 expression using GO terms and KEGG pathways. Based on *p*.adj <0.05 and *q* value <0.2, a total of 321 biological processes, 101 cellular components, 48 molecular functions, and 11 KEGG annotations were associated with NPM1 co-expressed genes. GO term notes indicate that these genes are mainly involved in ribosome biogenesis, cytosolic part and catalytic activity, acting on RNA. KEGG pathway studies showed that these genes were significantly associated with Ribosome, RNA transport and Spliceosome. The above results were shown by bubble plots (Figures 4A–D).

### The relationship between nucleophosmin 1 and tumor immune infiltrating cells in gastrointestinal cancers

**FIGURE 5:**
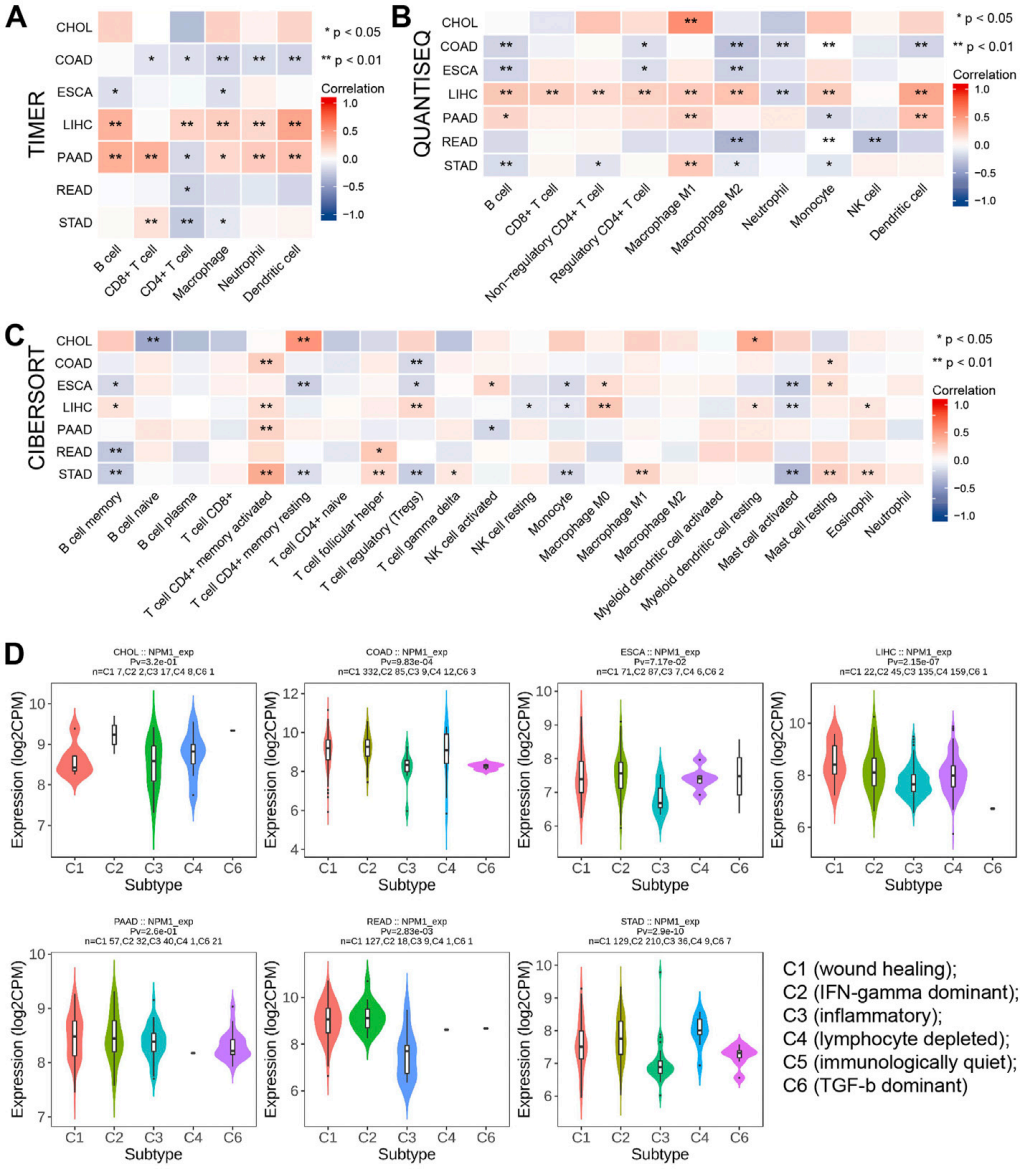
The relationship between NPM1 and tumor immune infiltrating cells in gastrointestinal cancers. **(A–C)** The correlation between NPM1 expression and immune cell infiltration was analyzed by TIMER, QUANTISEQ and CIBERSORT algorithm. **(D)** TISIDB analysis showed the association between NPM1 expression level and 5 immune subtypes.

A TIMER analysis revealed a negative correlation between NPM1 and the expression of five immune cells in COAD (CD8^+^ T-cells, CD4^+^ T-cells, macrophages, neutrophils, and dendritic cells). LIHC results showed a positive correlation between NPM1 expression and the expression of five immune cells (CD4^+^ T-cells, B cells, macrophages, neutrophils, and dendritic cells). Positive correlations were observed between NPM1 and five immune cells (CD8^+^ T-cells, B cells, macrophages, neutrophils and dendritic cells), while negative correlations were observed between NPM1 and CD4^+^ T-cells in PAAD (Figure 5A). QUANTISEQ analysis showed that NPM1 was negatively correlated with the expression of six immune cells (B cells, Regulatory CD4+T-cells, Macrophages M2, Neutrophils, Monocytes and Dendritic cells) in COAD. In LIHC, NPM1 was positively correlated with the expression of 8 immune cells (B cells, CD8+T-cells, Non-regulatory CD4+T-cells, Regulatory CD4+T-cells, Macrophages M1, Macrophages M2, Monocytes and Dendritic cells), and negatively correlated with the expression of Neutrophils, which was similar to the result of TIMER analysis (Figure 5B). However, CIBERSORT analysis showed that NPM1 was associated with the expression of a variety of different immune cells in gastrointestinal cancer (Figure 5C). However, TISIDB analysis showed that NPM1 expression was significantly enriched in five immune subtypes, namely wound healing, IFN-gamma dominant, inflammatory, lymphocyte depleted, and TGF-β dominant, in COAD, LIHC, READ, and STAD, but not in CHOL, ESCA, and PAAD (Figure 5D).

### Correlation between nucleophosmin 1 with m6A related genes in gastrointestinal cancer

**FIGURE 6:**
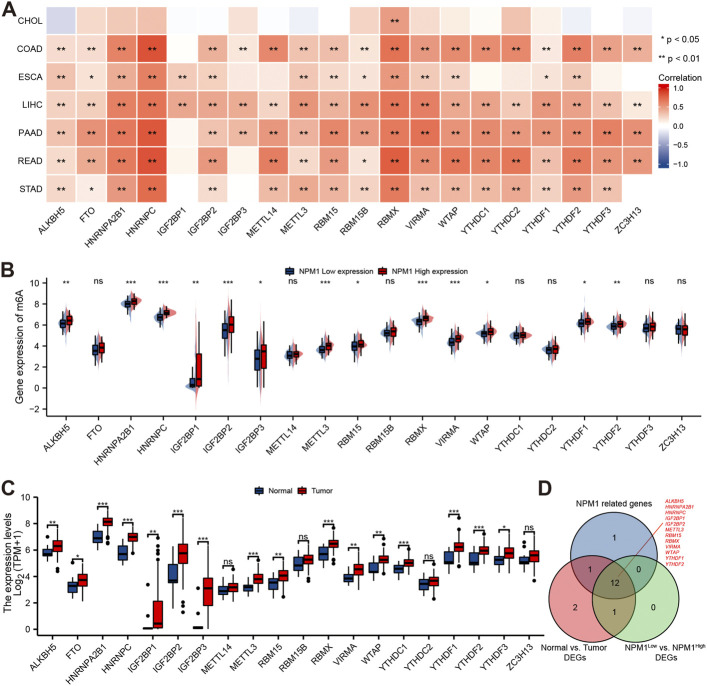
Correlation between NPM1 with m6A related genes in gastrointestinal cancer. **(A)** Correlation between the expression levels of NPM1 and m6A related genes in gastrointestinal cancer. **(B)** Differences in m6A related gene expression in ESCA cohorts based on low and high NPM1 expression. **(C)** ESCA cohort m6A related gene expression differs between tumor and normal groups. **(D)** Veen map shows overlapping genes.

A study conducted using the TCGA database explored the relationship between NPM1 and 20 m6A related genes expressed in gastrointestinal cancers in the CHOL, COAD, ESCA, LIHC, PAAD, READ, and STAD cohorts. In the CHOL cohort, NPM1 was not correlated with most m6A related genes as shown in Figure 6A. However, in the COAD, ESCA, LIHC, PAAD, READ, and STAD cohort, NPM1 was associated with the expression of most m6A related genes. ESCA cohorts were grouped based on expression levels of NPM1. A high NPM1 expression group had higher ALKBH5, HNRNPA2B1, HNRNPC, IGF2BP1, IGF2BP2, IGF2BP3, METTL3, RBM15, RBMX, VIRMA, WTAP, YTHDF1, and YTHDF2 expression than a low NPM1 expression group (Figure 6B). According to our analysis of differentially expressed m6A related genes among tumors and normals, ALKBH5, FTO, HNRNPA2B1, HNRNPC, IGF2BP1, IGF2BP2, IGF2BP3, METTL3, RBM15, RBMX, VIRMA, WTAP, YTHDC1, YTHDF1, YTHDF2, and YTHDF3 were expressed higher in tumors (Figure 6C). Finally, we screened 12 key genes (ALKBH5, HNRNPA2B1, HNRNPC, IGF2BP1, IGF2BP2, METTL3, RBM15, RBMX, VIRMA, WTAP, YTHDF1, and YTHDF2) according to the expression correlation and group expression differences (Figure 6D).

**FIGURE 7:**
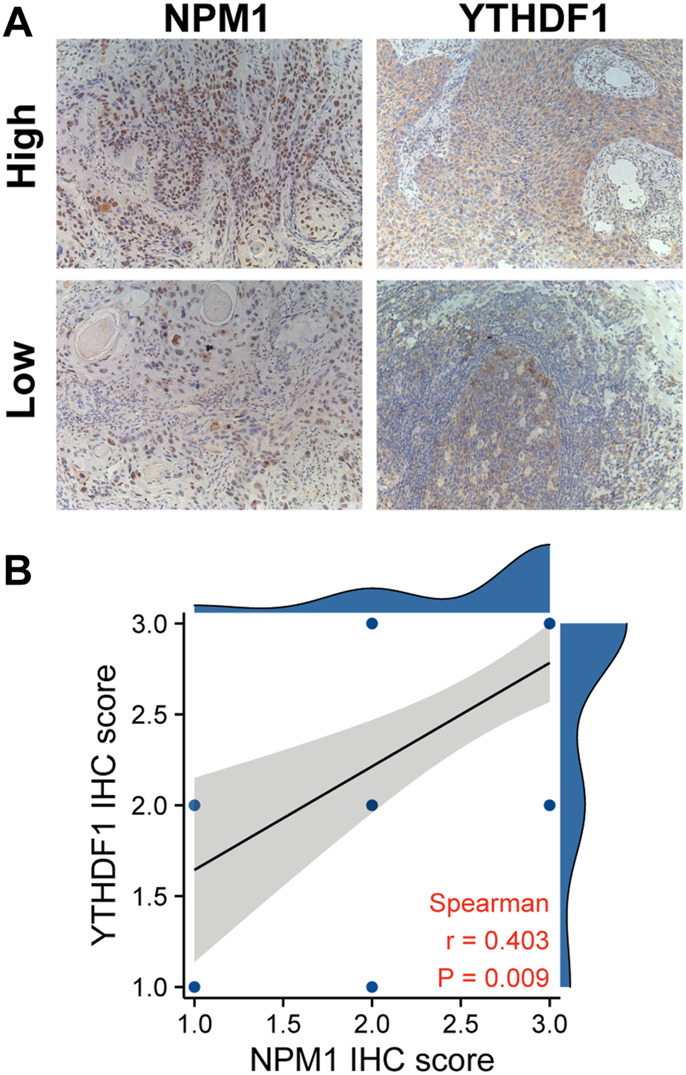
NPM1 was positively correlated with YTHDF1 IHC score in ESCA. **(A)** IHC picture of NPM1 and YTHDF1 in ESCA. **(B)** NPM1 was positively correlated with YTHDF1 IHC score.

We further verified the correlation between NPM1 and m6A related gene expression by analyzing the IHC score data of NPM1 and YTHDF1. Results as shown in Figure 7, the IHC score of NPM1 was positively correlated with YTHDF1 in ESCA samples (*r* = 0.403, *p* = 0.009).

### Correlation between nucleophosmin 1 with cuproptosis related genes in gastrointestinal cancer

**FIGURE 8:**
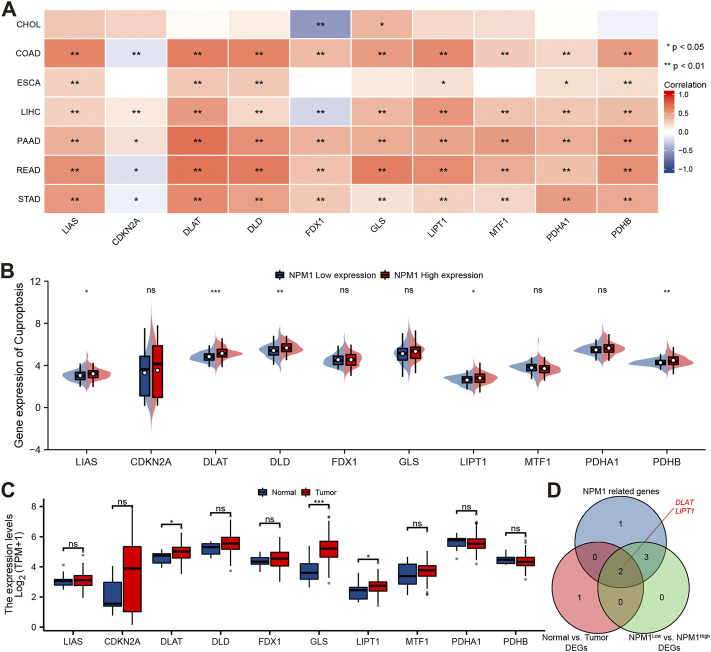
Correlation between NPM1 with cuproptosis related genes in gastrointestinal cancer. **(A)** Correlation between the expression levels of NPM1 and cuproptosis related genes in gastrointestinal cancer. **(B)** Differences in cuproptosis related gene expression in ESCA cohorts based on low and high NPM1 expression. **(C)** ESCA cohort cuproptosis related gene expression differs between tumor and normal groups. **(D)** Veen map shows overlapping genes.

A study conducted using the TCGA database explored the relationship between NPM1 and 10 cuproptosis related genes expressed in gastrointestinal cancers in the CHOL, COAD, ESCA, LIHC, PAAD, READ, and STAD cohorts. In the CHOL cohort, NPM1 was not correlated with most cuproptosis related genes as shown in Figure 8A. However, in the COAD, ESCA, LIHC, PAAD, READ, and STAD cohort, NPM1 was associated with the expression of LIAS, DLAT, DLD, LIPT1, PDHA1, and PDHB. In the ESCA cohort, we grouped according to the expression of NPM1. LIAS, DLAT, DLD, LIPT1, and PDHB expression was higher in the high NPM1 expression group than in the low NPM1 expression group (Figure 8B). According to our analysis of differentially expressed cuproptosis related genes among tumors and normals, DLAT, GLS, and LIPT1 were expressed higher in tumors (Figure 8C). Finally, we screened 2 key genes (DLAT and LIPT1) according to the expression correlation and group expression differences (Figure 8D).

## Discussion

Currently, NPM1 research is focused more on cancer than blood diseases. Zhou et al. found that NPM1 was overexpressed in LIHC cells, and high NPM1 expression was significantly associated with decreased survival and increased recurrence in LIHC patients (Zhou et al., 2017). Liu et al. (2012) found that the overexpression of NPM1 in COAD cells enhanced cell migration and invasion, and improved cell proliferation. Although it has been confirmed that overexpression of NPM1 in a variety of gastrointestinal cancers can promote the proliferation and migration of tumor cells, no comprehensive analysis of NPM1 in gastrointestinal cancers has been reported.

In this study, we found that NPM1 was overexpressed in gastrointestinal cancer through the analysis of UCSC XENA database and GEO database, which was consistent with some previous research results (Liu et al., 2012; Zhu et al., 2015; Matsui et al., 2022). At the same time, At the same time, we verified the overexpression of NPM1 in ESCA by cell experiments and IHC. We also found that the expression level of NPM1 is highly accurate in the diagnosis of gastrointestinal cancer (AUC >0.75), and the overexpression of NPM1 is related to the low survival rate of some patients with gastrointestinal cancer, indicating that the clinical correlation between NPM1 and gastrointestinal cancer is significant.

In further functional exploration, we found that 385 genes were positively correlated with NPM1 expression in 7 gastrointestinal cancers. According to the sum of the correlations of these 385 genes in gastrointestinal cancer, we screened the 30 genes with the strongest correlations, which are RARS1, BTF3, HSPA4, NSA2, HNRNPA1, EIF3M, CCT4, OLA1, UIMC1, G3BP1, NACA, RSL1D1, ETF1, MRPS27, RBM22, CCT8, GEMIN5, LARS1, PA2G4, CDK7, NIFK, RSL24D1, EIF3E, THG1L, INTS13, UBE2D2, RPS23, DIMT1, RIOK2, and TTC27 respectively. Previous studies have reported that overexpression of BTF3, CCT8, CDK7, ELF3M, G3BP1, HSPA4, OLA1, and RSL1D1 can contribute to the occurrence and development of CRC (Goh et al., 2011; Zhou et al., 2019; Zhou et al., 2021b; Liao et al., 2021; Zhang et al., 2021; Liu et al., 2022a; Liu et al., 2022b; Li et al., 2022), overexpression of CCT4 and PA2G4 can contribute to the occurrence and development of LIHC(Li et al., 2021a; Sun et al., 2022), overexpression of DIMT1 and HNRNPA1 can contribute to the occurrence and development of STAD (Liu et al., 2017; Zhu et al., 2022), and overexpression of EIF3E and UIMC1 can contribute to the ESCA occurrence and development (Xu et al., 2018; Yang et al., 2018). Therefore, we believe that these genes have the potential to be called diagnostic and therapeutic targets of gastrointestinal cancer. However, studies on the interaction between NPM1 and these genes in gastrointestinal cancer have not been reported. We will continue to pay attention to this interesting phenomenon in the follow-up research. In further enrichment analysis, we found that the co expression of NPM1 was mainly related to Ribosome Biogenesis, Cytosolic Part and Catalytic Activity, Acting On RNA. According to KEGG pathway analysis, NPM1 co-expression is primarily associated with Ribosomes. It has been found that the above biological functions and pathways play an important role in the occurrence and development of tumors, according to some scholars (Ruggero and Pandolfi, 2003; Ding et al., 2022). These findings suggest that NPM1’s co-expression network plays a critical role in the formation and progression of tumors.

The components of TME are complex, in which immune regulation and immune escape are important components. More and more studies have shown that TME plays a key role in tumor progression (Wang et al., 2020b; Chamma et al., 2022; Malla et al., 2022). In this study, we found that the overexpression of NPM1 was negatively correlated with most immune infiltrating cells in COAD and ESCA, but positively correlated with immune infiltrating cells in LIHC and PAAD. It was also found that NPM1 expression was significantly enriched in five immune subtypes in COAD, LIHC, READ and STAD. Helmink et al., 2020 Found that B cells can secrete a series of cytokines, which may potentially promote the anti-tumor response by producing antibodies to tumors. The lack of B cell infiltration will affect the inhibitory effect of the immune system on tumor cells (49). Denardo and Ruffell (2019) Found that in the initial stage of tumor development, macrophages can directly promote anti-tumor response by killing tumor cells. Additionally, there was a negative correlation between NPM1 expression and macrophage and B cell infiltration in ESCA. We believe that patients with gastrointestinal cancer express high levels of NPM1 which may result in immune escape and anti-tumor immunity, suggesting that NPM1 may be important in regulating the immune response to gastrointestinal cancers.

M6A modification is a reversible dynamic RNA epigenetic process, which is regulated by m6A regulatory factors and is crucial in the development of cancer (Chen et al., 2019; Li et al., 2019; Wang et al., 2020a). In gastrointestinal cancers, however, there has been no research on NPM1 and m6A related genes. In this study, we found that most gastrointestinal cancers expressed NPM1 positively correlated with m6A related genes. Especially in the ESCA cohort, we screened 12 key genes, ALKBH5, HNRNPA2B1, HNRNPC, IGF2BP1, IGF2BP2, METTL3, RBM15, RBMX, VIRMA, WTAP, YTHDF1, and YTHDF2, according to the expression correlation and group expression differences. It has been reported that the overexpression of ALKBH5(Nagaki et al., 2020), HNRNPA2B1 (Li et al., 2021b), HNRNPC(Zhang et al., 2019), IGF2BP1(Fang et al., 2021), IGF2BP2(Huang et al., 2021), METTL3 (Liu et al., 2020), WTAP (Zhu et al., 2021) and YTHDF1 (Liu et al., 2022c) can contribute to the occurrence and development of ESCA through different regulatory methods. At the same time, the correlation analysis of IHC score also confirmed that the expression of NPM1 was positively correlated with YTHDF1. Therefore, it is believed that the cancer-promoting activity of NPM1 gene is related to the expression of genes related to m6A. It is possible that NPM1 may be able to influence the methylation level of ESCA by affecting m6A, and ultimately affect the development of cancer.

Copper is the basic element to maintain human life activities, and plays an essential role as a cofactor of essential enzymes. Compared with normal cells, cancer cells have a higher demand for copper (Cobine and Brady, 2022; Tang et al., 2022; Tsvetkov et al., 2022). However, the relationship between NPM1 and cuproptosis related genes in gastrointestinal tumors has not been studied. A significant correlation was found in this study between the expression level of NPM1 and the expression level of cuproptosis related genes in most gastrointestinal cancers. In the ESCA cohort, we screened two key genes, DLAT and LIPT1, based on expression correlation and group expression differences. Goh et al. Found that DLAT is highly expressed in STAD, and interfering with the expression of DLAT can inhibit the proliferation of STAD cells (Goh et al., 2015). Lv et al. Found that LIPT1 expression increased in skin cutaneous melanoma biopsy and was an independent prognostic indicator of skin cutaneous melanoma patients (Lv et al., 2022). Accordingly, we believe that the cancer-promoting effect of NPM1 gene is associated with cuproptosis related genes expression. The NPM1 may adversely affect ESCA cells’ cuproptosis process by altering copper ion levels, which ultimately affects cancer development.

**FIGURE 9:**
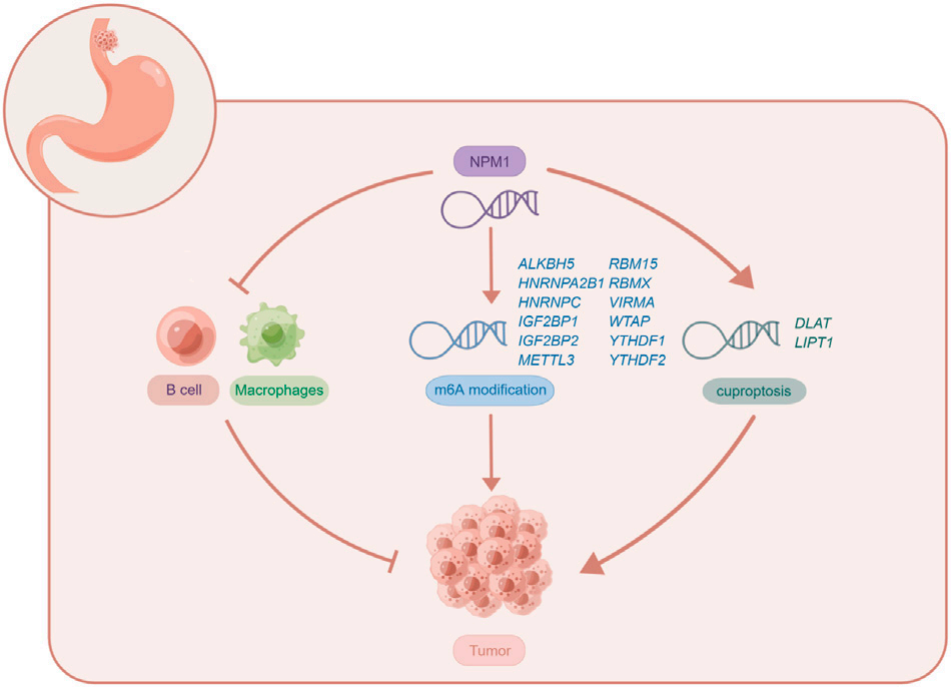
The potential biological function of NPM1 in ESCA. Figures were created by Figdraw (www.figdraw.com).

In conclusion, this is the first comprehensive study to examine NPM1 expression in relation to m6A, cuproptosis and tumor cell immune infiltration in gastrointestinal cancer. This study shows that NPM1 is highly expressed in gastrointestinal cancers, and its expression level can accurately diagnose cancer, and is a prognostic indicator of ESCA, LIHC, and PAAD. The expression of NPM1 is negatively correlated with B cell and macrophages infiltration, which may affect the tumor immunity of ESCA by affecting B cell and macrophages infiltration. NPM1 is closely related to 12 m6A related genes, which may affect the tumor progression of ESCA by affecting the methylation level of m6A. However, NPM1 is also positively correlated with the expression of two cuproptosis related genes, which may affect the regulation of cuproptosis in tumor cells by affecting the expression of these genes. The potential biological function of NPM1 in ESCA is shown in Figure 9. Of course, further exploration is needed to confirm the function and potential mechanism of NPM1 in the occurrence and development of gastrointestinal cancer.

## Data Availability

The datasets presented in this study can be found in online repositories. The names of the repository/repositories and accession number(s) can be found in the article/Supplementary Material.
