# Supplementary material for: Nucleophosmin 1 is a prognostic marker of gastrointestinal cancer and is associated with m6A and cuproptosis
Source: Front Pharmacol. 2022 Sep 14;13:1010879. doi: 10.3389/fphar.2022.1010879 (PMC9515486; doi:10.3389/fphar.2022.1010879)
Supplement: Supplementary file 1 [file DataSheet1.docx]

Supplementary Material

# Supplementary Methods

## Immunohistochemistry (IHC)

ESCA samples were obtained from 41 patients treated surgically at Taihe Hospital Affiliated of Hubei University of Medicine between 2018 and 2020. In short, tissue sections were incubated overnight with antibodies against NPM1 (1:250, Proteintech) and YTHDF1 (1:150, Proteintech), then incubated with HRP secondary antibodies (1:500, Abcam), then stained with DAB, and finally counterstained with hematoxylin.

IHC staining scores of NPM1 and YTHDF1 were assessed by two experienced observers. IHC score of tumor cells was 0-3: 0, negative; 1, weak; 2, medium; 3, strong.

## Cell cultures and qRT-PCR

Human ESCA cell lines ECA109 and KYSE-150 and normal human squamous esophageal cell line Het-1A were obtained from the American Type Culture Collection (Manassas, VA, USA). The cells were maintained in DMEM high glucose medium (Hyclone, Logan, UT, USA) supplemented with 10% FBS (Gibco, USA) and 1% antibiotics (penicillin-streptomycin, Gibco, USA).

Total RNA was isolated from cells using Trizol reagent (Invitrogen, Carlsbad, CA, USA). Use Prime Script RT reagent kit (Takara, Dalian, China) for reverse transcription, and then use SYBR Prime Script RT PCR kit (Takara, Dalian, China) for qRT-PCR. Use ACTB as an internal reference and use the 2^-△△Ct^ method to calculate the results. NPM1 primer sequences: forward primer CGCTGTGGAGGAAGATGCAG and reverse primer GGCAGACCGCTTTCCAGATA. ACTB primer sequences: forward primer TCTTCCAGCCTTCCTTCCT and reverse primer AGCACTGTGTTGGCGTACAG.
